# Effect of Gamma Irradiation on the Structural, Optical, Electrical, and Ferroelectric Characterizations of Bismuth-Modified Barium Titanate Ceramics

**DOI:** 10.3390/ma15124337

**Published:** 2022-06-19

**Authors:** Hanan Al-Ghamdi, Aljawhara H. Almuqrin, Hamoud Kassim

**Affiliations:** 1 Department of Physics, College of Science, Princess Nourah Bint Abdulrahman University, Riyadh 11671, Saudi Arabia; hmalghmdi@pnu.edu.sa (H.A.-G.); ahalmoqren@pnu.edu.sa (A.H.A.); 2 Department of Physics and Astronomy, College of Science, King Saud University, Riyadh 11451, Saudi Arabia

**Keywords:** gamma irradiation, structural properties, octahedral distortion, bandgap energy, remnant polarization

## Abstract

Materials with ferroelectric properties, low bandgap energies, high polarization, low loss, and thermal stability are essential for future solar-cell applications. Researchers have attempted to obtain such materials by using several approaches. In this vein, a novel approach is reported in this work using gamma ray irradiation. The effect of gamma radiation on the structural, optical, and ferroelectric characterizations of bismuth (Bi)-doped barium titanate (BaTiO_3_ (BT)), namely Ba_0.95_Bi_0.05_TiO_3_ ceramics (abbreviated as (Bi:BT)), was investigated. X-ray diffraction, structure refinement, and Raman study revealed the presence of a perovskite structure with a tetragonal phase in all investigated samples. Morphological study revealed a nonuniform grain size and some porosity. Gamma irradiation-induced combined effects were proved by a detailed analysis of bond lengths, bond angles, octahedral distortions, oxygen vacancies, and charge compensations. Electron paramagnetic resonance (EPR) study gave direct evidence of oxygen vacancies in the irradiated samples. After gamma irradiation, UV–vis study indicated a decrease in the bandgap from 3.14 to 2.80 eV and a significant increase in visible light absorption. Cole–Cole plots confirm as an increase in gamma-ray dose results in higher levels of electron hopping. Study of the P–E hysteresis loop demonstrated that ferroelectric properties could be maintained after gamma irradiation, with a slight decrease in remnant polarization. The behaviour of the P–E was correlated with increasing gamma dose in the investigated ceramics, demonstrating a strong gamma dependence in the loops’ profile. We guess that the present approach may be a promising technique for enhancing the multifunctionality of electronic devices.

## 1. Introduction

Researchers have been intrigued by the extraordinary ferroelectric behaviour of barium titanate. An impressive ferroelectric material with remarkable properties, it is well suited for use in actuators, sensors, ultrasonic transducers, resonators, filters, dielectric capacitors, etc. because of its high dielectric constant, good ferroelectric properties, and significant optoelectronic properties [1,2,3,4,5]. Additionally, BaTiO_3_ is commonly used multilayer ceramic capacitor (MLCC) fabrication [6,7], homographic memories [8], waveguide modulators [9,10], IR detectors [11] and gate dielectrics [12]. Barium titanate (BT) is a member of the perovskite ABO_3_ structure, where Ba^2+^ occupies the corner A-site, Ti^4+^ occupies the centre B site, and O^2−^ occupies the face centre. The BT structure can be viewed as a TiO_6_ octahedron surrounded by Ba^2+^ atoms [1]. The physical properties of barium titanate can be tailored by using different approaches such as processing conditions [13] and doping processes [14]. Chemical modification, including chemical doping, is the most utilized approach for tailoring barium titanate’s ferroelectric, dielectric, and optical properties [15,16,17,18]. Chemical doping on the host lattice can give rise to lattice distortion, octahedral tilting, and the creation of defects [19]. This results in a change in the homogeneity, phase structure, and grain size. From one point of view, there is a possibility of using barium titanate-based materials in environments of nuclear radiation, e.g., as sensors for reactors, particle accelerators, spaceships, satellites, detectors, etc. [20]. Rapid heavy ion irradiation and swift ion irradiation have been reported as techniques effective in modifying the microstructures of BaTiO_3_ ceramics [20,21,22]. A wide variety of defect states are created in a material after being exposed to energetic ions. These alter the material’s structural, optical, and electrical properties [23,24,25]. These changes depend strongly on the mass of the incident ion, the energy of the irradiation, and the radiation fluence [26]. In the presence of gamma radiation, the properties of materials that are structurally dependent may be altered, and hence, their performances may be affected. These changes are highly dependent on radiation parameters such as linear energy transfer, dose, and energy, as well as material properties and structure [27]. There have been very few studies on the effect of gamma radiation on BT-based ceramics, and an extensive literature search showed that no studies have been documented on gamma-ray irradiated Bi:BT ceramics. Therefore, to understand the effect of irradiation on the structural, electric, and optical properties of Bi:BT ceramics, it is essential to conduct extensive research. We report the effect of gamma irradiation on the microstructure of Bi:BT ceramics. These findings may provide important insights into the stability of devices constructed using BaTiO_3_ in radiation and space environments. A low melting oxide such as Bi_2_O_3_ used as a donor doping in the Ba-site can improve the sinterability of BaTiO_3_ ceramics, and the sintering temperature of barium titanate can be reduced. Solubility limits of Bi^3+^ in BaTiO_3_ have been reported of up to 5% [28,29]. During the charge compensation process, barium vacancies are formed, which enhanced solid-state mobility and thus reduced the sintering energy requirement. Thus, we doped 5% of Bi to substitute the Ba site of BaTiO_3_ ceramics. The novelty of this study was to combine the effects of both charge compensations induced by Bi^3+^ doping and gamma irradiation on the physical properties of BaTiO_3_ ceramics. The obtained results with different gamma doses proved that the optical properties were severely affected. This might have resulted in a decrease in the bandgap energy of the irradiated samples and an increase in the absorption spectrum in the visible range. This research aimed to check whether the increased oxygen vacancy concentration induced by gamma radiation could alter the absorption spectra and tune the bandgap energy of barium titanate modified by Bi. The tuning of bandgap energy via the octahedral distortion and oxygen vacancies induced by gamma irradiation is a new approach that opens the door to researchers developing optoelectronic devices to manipulate the bandgap in ferroelectric materials.

## 2. Experimental Part

Polycrystalline Ba_0.95_Bi_0.05_TiO_3_ (Bi:BT) ceramics was fabricated by the solid-state reaction technique. High-purity powders of BaCO_3_, Bi_2_O_3_, and TiO_2_, with purities of 99.99%, 99.97%, and 99.8%, respectively (Sigma-Aldrich, St. Louis, MI, USA), were used as starting materials. In order to offset the volatilization of bismuth, a 2 mol% Bi_2_O_3_ excess was added. The starting powders were mixed with acetone in an agate mortar. A liquid, i.e., acetone, was utilized as part of the dispersed phase during the mixing process. The powder was blended and bonded with acetone, which served as a lubricant during milling for obtaining powder in a homogeneous manner. Consequently, the mixed powder dried in an oven at 50 °C for 1 h. The dried mixture was heat treated in an oven at 1000 °C for 6 h. The heat-treated powder was ground in acetone for a duration of 8 h using a high-energy ball mill. Green pellets, each with a 1 mm thickness and 10 mm diameter, were prepared using 1% by weight of polyvinyl alcohol (PVA) as a binder. The sintering conditions in the first stage were prolonged heating to 500 °C at a heating rate of 2 °C/min until the binder was removed. In the second stage of sintering, from 500 °C to 1250 °C, the process was carried out at a 5 °C/min heating rate. Then, the temperature was held constant at 1250 °C for 4 h for structure densification. After that, the temperature decreased to room temperature at a cooling rate of 5 °C/min. In this experiment, we applied the γ-ray with different doses, namely 0 Gy, 400 Gy, 800 Gy, and 1000 Gy, using a cobalt-60 gamma source (T1/2 = 5.27 year) (Nordion, model GC-220). Phase detection was performed using a Bruker D8 powder X-ray detector. The average grain size of the sintered sample was measured using the ImageJ software. Phase detection was also performed with a micro-Raman spectrometer (Wi-Tec) using an Nd:YAG laser with a wavelength of 532 nm as the excitation source. Microstructural analysis was performed using a Carl Zeiss Ultra 55 field emission Scanning electron microscope (FE-SEM). At room temperature, Bruker Elexsys E580 spectrometers were used to measure the EPR spectra operating at X-band (≈9.5 GHz). Optical characterization of the samples at room temperature was performed using a UV-2600 (Shimadzu UV–vis spectrophotometer) (UV-2600; Shimadzu, Kyoto, Japan) in the wavelength range from 200 to 1400 nm. An impedance analyser was used to determine the Z″ and Z′ of the samples. At room temperature, a modified Sawyer–Tower circuit was used for ferroelectric measurements at a 10 Hz frequency.

## 3. Results and Discussion

**Figure 1:**
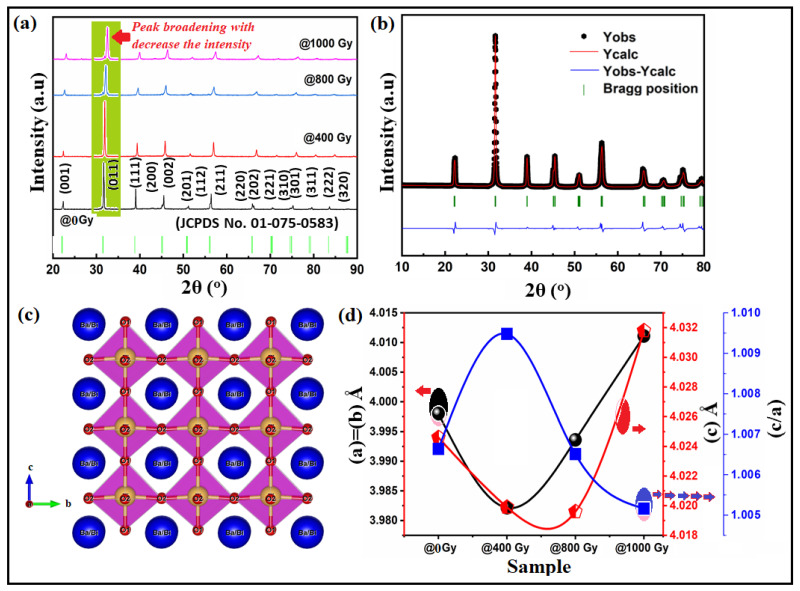
(**a**) XRD pattern of Bi:BT ceramic before (black line) and after gamma irradiation (coloured lines) measured at room temperature; (**b**) the XRD refinements of Bi:BT ceramic, with the observed data represented by black circles, the refinement profile by pink, and the difference between measured and calculated diffraction patterns by blue. P4mm Bragg reflections are shown in green; (**c**) a schematic of the crystal structure of the Bi:BT ceramics, blue circles represented the Ba/Bi atoms, yellow circles represented the Ti atoms and the red circles represented the oxygen atoms; (**d**) the variation in the lattice constants (a-parameter in black line) & c-parameter in red line) and tetragonality (c/a in blue line) with gamma dose.

**Figure 2:**
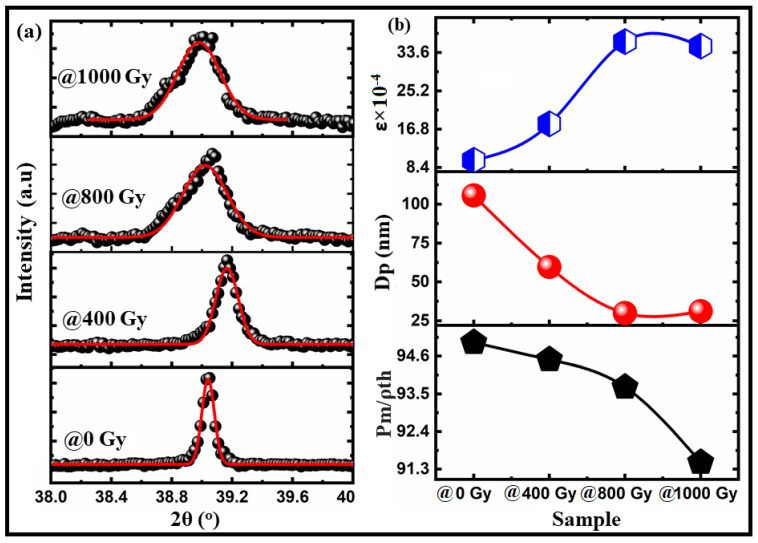
(**a**) A Gaussian fitting of the (111) plane for Bi:BT ceramics irradiated with different gamma doses; (**b**) the variation in crystallite size, lattice strain, and relative density of Bi:BT ceramics irradiated with different gamma doses.

Figure 1a shows the XRD patterns of Ba_0.95_Bi_0.05_TiO_3_ (Bi:BT) ceramics measured at room temperature before and after gamma irradiation. The observed patterns revealed that all samples, before and after the irradiation, had a single tetragonal phase without any secondary phase. The XRD patterns of all samples were indexed based on the JCPDS database and found to correspond well to standard BaTiO_3_, which also has a tetragonal structure (JCPDS No. 01-075-0583) [30]. In the analysis of the obtained results for irradiated ceramics, no evidence of secondary phases was received, indicating that neither gamma irradiation nor defects caused by strong structure disordering resulted in secondary phases. Examining the X-ray diffraction patterns revealed that the main variation seemed to be associated with peak intensities and peak broadening that occurred because of an external influence such as lattice distortion. At the same time, the greatest variation in diffraction patterns was seen for the samples irradiated with doses above 400 Gy. These not only showed reduced intensity but shifted towards the higher 2θ side, which indicated a decrease in the unit cell volume as shown in Figure 1d. As per the literature reports, variation in the structural parameters and volume of the crystal lattice can be caused by the interpretation of the crystal lattice deformation. In the irradiation case, the deformation increases or decreases because of tensile stresses. As a result, the parameters and volume of the crystal lattice may increase or decrease accordingly [31]. To verify the correctness of the lack of influence of the crystal phase of the Bi:BT sample after radiation, the X-ray data were subjected to Rietveld refinement analysis using the Fullprof software, as shown in Figure 1b. The accuracy of the investigated structure was judged by examining the resulting values of the R-factors R_wp_, R_b_, R_exp_, and χ^2^. A plot difference between the observed and calculated patterns was also used to evaluate the quality of the Rietveld refinement. The bond lengths and angles were also estimated from this refinement and summarized in Table 1. Interestingly, the Ti-O(1) and Ti-O(2) bond lengths decreased after gamma irradiation. Furthermore, the O-Ti-O covalent bond was displaced in the perovskite lattice, which could have been due to the octahedral distortion TiO6 induced by gamma irradiation. The distortion of the octahedral structure may have been a consequence of oxygen vacancies being introduced into the BT lattice by the doped trivalent ions of Bi into the Ba^2+^ site. A schematic representation of the Bi:BT ceramic unit cell was designed using the VESTA program and is presented in Figure 1c. To assess the variation in the FWHM, which characterized the distortion of the lattice, a Gaussian fit was performed on the peak of the plane (111), as shown in Figure 2a. We applied the Scherrer and Williamson–Hall formulas to determine the average crystallite size and lattice strain using the FWHM obtained from this fitting [32]. The obtained results are depicted in Figure 2b. According to the obtained data, in the case of the sample with radiation > 400 Gy, variation in crystallite size and lattice strain was observed, which proved an increase in the distortion in the crystal lattice. On the other hand, Bi ion doping in the BaTiO_3_ lattice had an effect. It was previously [29,33] reported that the positive charge of oxygen vacancies would compensate for the presence of negatively charged Ba^2+^-vacancies in nondoped BaTiO_3_. Compared with the ideal BaTiO_3_ crystal, this defect can shrink the unit cell. Bi^3+^ ions were incorporated into vacant Ba^2+^ positions, and oxygen vacancies were simultaneously eliminated, as shown by the Kroger–Vink notation

$${\mathrm{Bi}}_{2}O_{3}\to 2{\mathrm{Bi}}_{\mathrm{Ba}}^{\cdot }+V_{\mathrm{Ba}}^{\prime \prime }+3O_{O}^{\times },$$

in which BaTiO_3_’s (a) and (c) parameters were increased.

Interestingly, Figure 1d clearly shows that the lattice parameters were expanded when the gamma ray dose was >400 Gy. This phenomenon was reported by Serkin D. Günay [34], who called it lattice swelling, which may be caused by defect intergrowth. Radiation damage accumulation may occur in the near-surface layer from gamma irradiation, causing defects such as vacancies. The defect accumulation can cause a decrease in the relative density of the samples, as shown in Figure 2b. Lattice distortion and defect accumulation had an effect on the optical and electrical properties of the investigated samples, as shown in the next sections. Formulas (1) and (2) were used to obtain the distortion of the crystal lattice based on changes in the lattice parameters of the crystal and characterize the gamma influence on the crystal lattice [31]:

(1) $$Distortion\;crystal\;lattice\;\left(a\right)=\frac{a_{irr}-a_{0}}{a_{0}}$$

(2) $$Distortion\;crystal\;lattice\;\left(c\right)=\frac{c_{irr}-c_{0}}{c_{0}}$$

where *a_irr_* and *c_irr_* are the lattice constants after gamma irradiation and *a*_0_ and *c*_0_ are the lattice constants before gamma irradiation. The obtained parameters are listed in Table 1. Similar observations were reported by Zhang et al. [35] and Liu et al. [36].

**Figure 3:**
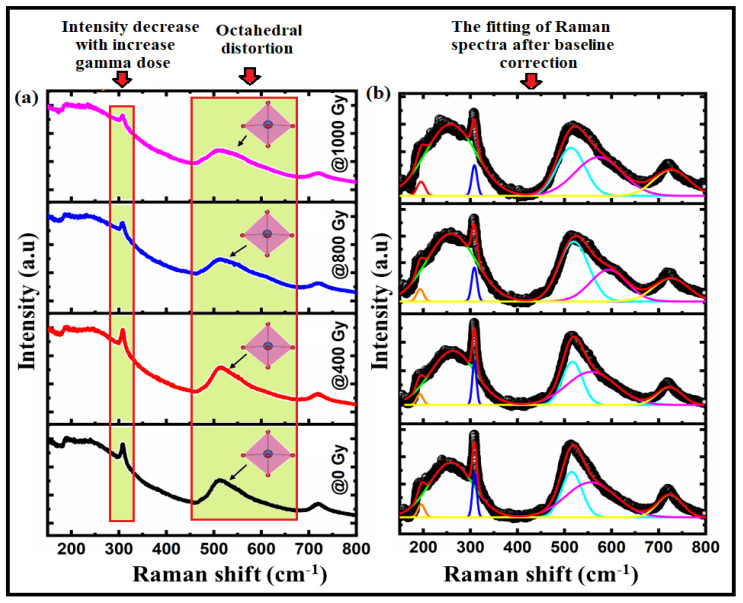
(**a**) The Raman spectra of Bi:BT ceramics before (black line) and after (red, blue, and pink lines) gamma irradiation; (**b**) the fitting of the Raman spectra (black color) to the Gaussian-Lorentzian function (red-color) measured at room temperature.

We used Raman spectroscopy to obtain sufficient information about the Raman modes in the Bi:BT samples before and after gamma irradiation, providing a structural fingerprint by which the phase structure could be identified [37,38]. Figure 3a shows the Raman spectra in the wavelength range 180–830 cm^−1^ of Bi:BT ceramics along with the corresponding spectral deconvolution in Gaussian peaks at room temperature. According to group theory, in Bi:BT before and after gamma irradiation, the A_1_(TO_2_) mode was embedded at 245 cm^−1^ and 244 cm^−1^, the sharp E(TO_3_) mode was at 309 cm^−1^ and 308 cm^−1^, the A_1_(TO_3_) mode was embedded at 512 cm^−1^ and 509 cm^−1^, and the A_1_(LO_3_) mode was at 724 cm^−1^ and 721 cm^−1^, which was consistent with observations in the literature [39]. Moreover, the Bi:BT ceramics appeared to exhibit additional modes in the spectra, such as the E(TO_2_) mode with wavenumbers around 197 cm^−1^ and 195 cm^−1^, which was associated with the shift of the Bi atoms on the Ba-O vibrational modes. The modes of A_1_(TO_2_), E(TO_3_), and E(LO_3_) were related to the shifting of Ti-O bonds. However, the modes of A_1_(TO_3_) and E(LO_4_) were related to the stretching or elongation of the TiO_6_ octahedra. Finally, the A_1_(LO_3_) mode was correlated with octahedral distortion in the ferroelectric phase [39,40,41]. The broadening of the A_1_(TO_3_) mode after irradiation was due to the overlap of the different modes [42]. Interestingly, most Raman modes shift to lower wavenumbers after gamma irradiation, which could be attributed to the shift of Ti cations or octahedral distortion [43,44,45,46] (see Figure 3 and Table 2). Furthermore, the decrease in the intensity of A_1_(LO_3_) mode after gamma irradiation confirmed the disorder caused by gamma irradiation [47].

**Figure 4:**
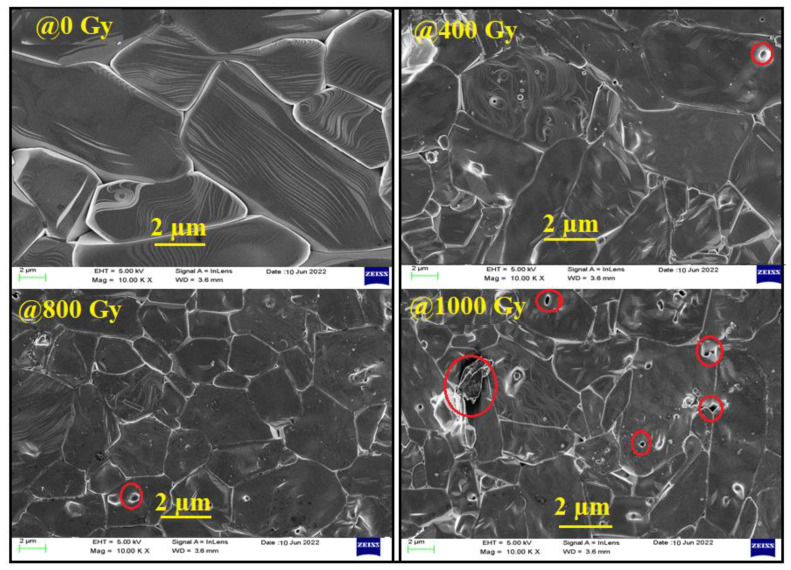
Scanning electron microscopy (SEM) of Bi:BT ceramics.

Figure 4 shows a microstructural comparison of Bi:BT ceramics before and after gamma irradiation. According to the figure, there was an uneven distribution of grains. Samples without irradiation consisted of many large grains and very few tiny grains. However, many small grains were present in the sample after irradiation. Some residual porosity could be seen in the sample after gamma irradiation. This result suggests that irradiation dosage at this level affected the uniformity of the composition. The average grain sizes of the samples were determined using the ImageJ software. The average grain sizes were found to be 6.977 μm, 5.303 μm, 4.755 μm, and 4.382 μm for the samples of 0 Gy, 400 Gy, 800 Gy, and 1000 Gy, respectively. The average grain size slightly decreased after exposure to a gamma dose. This variation in the average grain size may have been due to fractured grains as a result of the irradiation effect [48]. Similar observations were reported by A.K. Nath et al. [20,49]. Some porosities were observed on the irradiated samples, and their ratios were estimated using the formula [50]:

(3) $$P\left(\%\right)=\left(1-\frac{\rho_{m}}{\rho_{th}}\right)\%$$

The obtained values are reported in Table 1. From recorded data in Table 1, we concluded that the decrease in grain size may have affected the remnant polarization, as shown in the ferroelectric study section.

**Figure 5:**
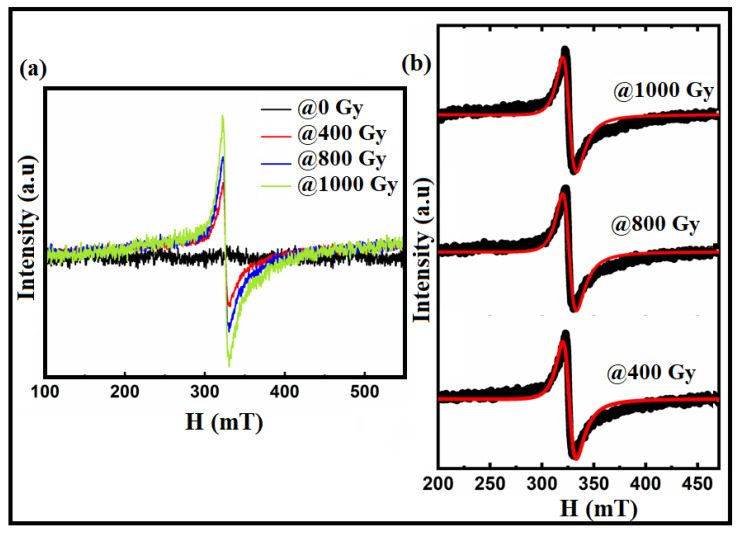
(**a**) EPR spectra of the Bi:BT samples before and after gamma irradiation; (**b**) the Lorentz fitting (in red lines) of the resonance spectra (in black color) for the irradiated samples at 400 Gy, 800 Gy, and 1000 Gy as measured at room temperature.

To examine the existence of oxygen vacancies in the materials at room temperature, electron paramagnetic Resonance (EPR) measurements were used. The EPR spectra of the Bi:BT samples before and after gamma irradiation measured at room temperature in the magnetic field range of 100 to 550 mT are shown in Figure 5. From Figure 5a, it is appearing that no resonance signal was observed in the sample before irradiation. However, in the irradiated samples, an asymmetrical line in the Lorentzian shape was observed and fitted using the Lorentz function as shown in Figure 5b. Using the parameters obtained from this fitting, such as magnetic resonance (*H_r_*), the Lande factor was calculated using the following equation [51,52]:

(4) $$g=\frac{hv}{\mu_{B}H_{r}}\;$$

where *H_r_* refers to the resonant field, *h* is Planck’s constant, *v* is the operating frequency (*v* = 9.5 GHz), and *μ_B_* is the Bohr magneton. The -factor, found to be in the range of 1.99 to 2.002, pointed to the existence of oxygen vacancies [53,54,55].

**Figure 6:**
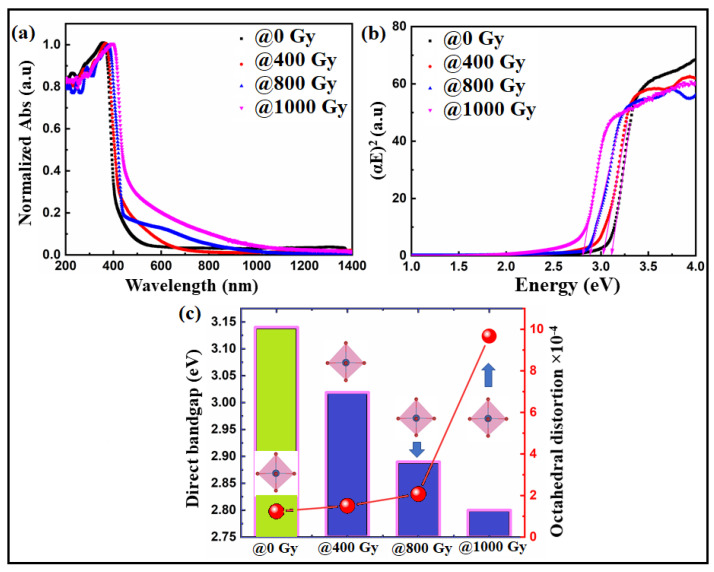
(**a**) UV–vis absorption spectra and (**b**) Tauc plot of the Bi:BT ceramics before (black line) and after (colored lines) gamma irradiation; (**c**) correlation between the bandgap and octahedra distortion in the Bi:BT ceramics before (green shadow) and after (blue shadow) gamma irradiation.

The absorption spectra of Bi:BT ceramics before and after irradiation were measured across the wavelength range of 200–1400 nm, as shown in Figure 6a. Prior to gamma irradiation, the sample showed an absorption edge in the ultraviolet range (410 nm), which agreed with a previous report on pure BT ceramics [56]. Interestingly, the absorption edge shifted significantly to higher wavelengths for the sample exposed to gamma irradiation, i.e., lower energies, as shown in the red curve. The direct bandgap energy of Bi:BT ceramics before and after gamma irradiation was determined using the Tauc plot [57]:

(5) $${\left(\alpha h\nu \right)}^{2}=A\left(h\nu -E_{g}\right)$$

where, in practice, the absorption coefficient is α, the energy of an incident photon is *hν*, and the bandgap energy is *E_g_.* We determined the direct bandgap from the intersection of the straight line with the X-axis by plotting (*αhν*)^2^ on the Y-axis against the energy of the incident photon (*hν*) on the X-axis of Figure 6b. The bandgap energies were found to be 3.14 eV, 3.019 eV, 2.89 eV, and 2.80 eV for the samples with gamma doses of 0 Gy, 400 Gy, 800 Gy, and 1000 Gy, respectively. The decreasing bandgap and the increased absorption spectrum in the visible region can be explained based on three factors: oxygen vacancies, charge compensation, and octahedral distortion. Oxygen vacancies may have increased in the Bi:BT ceramics as a result of the production of displaced atoms by exposure to gamma-ray radiation. Thus, in ceramics exposed to radiation, an increase in oxygen vacancies could result from two mechanisms: dissociation of dipolar complexes that release oxygen vacancies and displacement of oxygen atoms at interstitial sites caused by Compton electron bombardment [58,59,60,61,62,63]. An oxygen vacancy would produce a deep or shallow donor energy level in the bandgap. Consequently, it would affect the Fermi level and thus the bandgap. Because of O-vacancies levels above the Fermi level in Bi:BT, the entire electronic structure may have been altered. We guess that the presence of O-vacancies in the band structure near the Fermi level decreased the effective bandgap [64,65]. Consequently, this mechanism enhanced the absorption spectra and decreased the bandgap energy of irradiated samples as illustrated in Figure 6a,b.

For trivalent Bi^3+^ substitution at the barium site (Ba^2+^), a charge compensation, either by cation vacancies on the A- or B-site (ionic compensation) or by electrons (electronic compensation), was induced that is possible to express as three mechanisms [66,67]:

$$4{\mathrm{Ba}}^{2+}+{\mathrm{Ti}}^{4+}\to 4{\mathrm{Bi}}^{3+}+V_{\mathrm{Ti}}$$

$$3{\mathrm{Ba}}^{2+}\to 2{\mathrm{Bi}}^{3+}+V_{\mathrm{Ba}}$$

$${\mathrm{Ba}}^{2+}\to {\mathrm{Bi}}^{3+}+\;e^{\prime }$$

Based on the above, the doping of Bi increased the intensity of the visible absorption effectively, as shown in the red curve in Figure 6a.

Finally, distortions in the TiO_6_ octahedra may also have been responsible for the narrowing in the bandgap energy. Quantitively, the values of the octahedral distortion of the Bi:BT samples before and after gamma irradiation were estimated using the formula [68,69,70,71,72]:

(6) $$\Delta d=(\frac{1}{6})\underset{n=1,6}{\sum }{\left[\left(d_{n}-\langle d\rangle \;\right)/\langle d\rangle \right]}^{2}$$

where d_n_ is the individual bond distance (Ti-O) and 〈d〉 is the average of the bond distances. The octahedral distortion values for all samples before and after gamma irradiation are reported in Table 3. The distortion increased as the gamma dose increased. Accordingly, this may have created localized electronic levels in the bandgap between the valence and conduction bands of the Bi:BT samples, thus decreasing the values of the bandgap, as shown in Figure 6c. A similar observation was recently reported by Alkathy et al. [71,72,73]. Table 3 shows that such a distortion in the octahedrons would be a result of the tilting of octahedrons under the influence of gamma irradiation. As a result of this rotation, the oxygen–titanium distance changed, causing deformation in the unit cell. Abnormal octahedral variation could also have been caused by increased vibrations of the lattice resulting from gamma irradiation, which would have added energy to the crystal. In some cases, these factors could affect the peak intensity of XRD diffraction and affect the number of diffracting particles on the plane.

**Figure 7:**
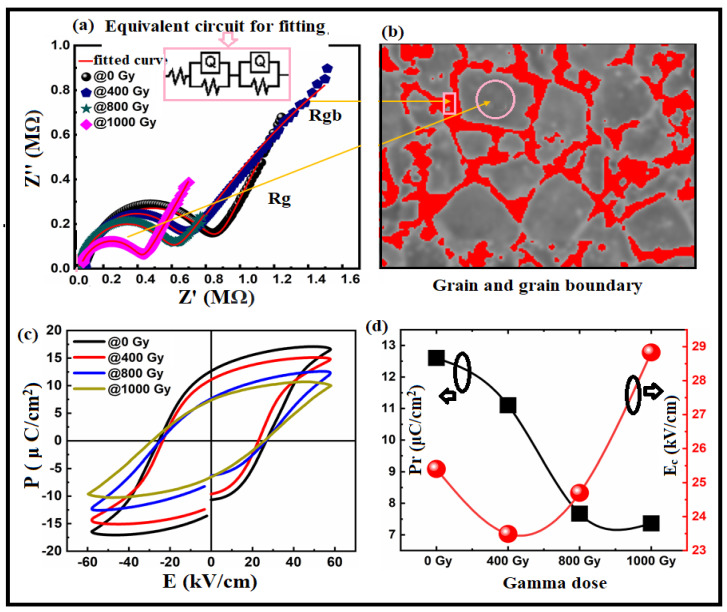
(**a**) Cole–Cole plots for the Bi:BT samples before and after gamma irradiation measured at room temperature and fitted using an equivalent circuit consisting of two RQ elements, as shown in the inset graph; (**b**) representation of the grain (grey) and grain boundary (red); (**c**) relationship between the polarization and electric field measured at 10 Hz for all samples before and after gamma irradiation at room temperature; (**d**) correlation between remnant polarization (in black line) and coercive field (in red line) with gamma dose irradiation.

By determining the contribution of grain boundaries and the grain boundaries themselves, impedance spectroscopy can be used to study the electrical heterogeneity of the sample. Under gamma irradiation, we investigated grain boundaries and grain resistance using complex impedance plots (Z′ vs Z″). Figure 7a depicts the complex impedance spectrum at room temperature. This figure demonstrates the distribution in relaxation times via the depressed semicircles. Since there were two or more relaxation times, an equivalent parallel circuit could represent the relaxation. In addition, parallel RQ circuits could be used, each representing a different relaxation time at a grain boundary or a grain contribution. The dotted samples represent the measured data, and the red line represents a parallel QR circuit model that fits the observed data. Two parallel RQ circuit models were used to fit the measured data. Figure 7a contains an inset with the equivalent circuit model. The RQ elements represent grain and grain boundary contributions and electrode effects [74]. High-frequency responses were related to the grain (bulk). Grain boundaries and electrode effects caused the low-frequency response. See Table 4 and Figure 7b for more information. From these results, we concluded that with gamma irradiation, conductivity changed profoundly when compared with impedance behaviour, resulting in a higher capacitance in the grain than the grain boundaries (Ref Table 4), as increasing dosage resulted in higher levels of electron hopping. The increase in electron hopping affected the behaviour of the hysteresis loop, as shown in Figure 7c. Semicircles at lower frequencies associated with grain boundaries, as proposed by Van Dijk et al. [75], were related to ionic conduction. Semicircles occurred at higher frequencies because of the electronic conduction contribution of the bulk of the grain.

Figure 7c shows the Bi:BT ceramic’s pre- and post-gamma irradiation ferroelectric P–E hysteresis loops. The measurements were performed at room temperature with a maximum 60 kV/cm field and 10 Hz frequency. As shown in the figure, there was an opening loop, which may have been due to the retention behaviour of the samples [76]. The Bi:BT ceramics showed a decrease in remnant polarization with increased gamma dose, as shown in Figure 7d. The coercive fields decreased when the gamma dose was 400 Gy and then increased thereafter. A decrease in Pr may have been related to the increasing oxygen vacancies at higher radiation doses. Furthermore, the grain size and porosity may have affected the hysteresis loop behaviour. According to [77,78,79], large grains exhibit larger remnant polarizations because domain switching occurs more readily in larger grains under external electric fields [79,80,81].

## 4. Conclusions

This work investigated the effect of gamma irradiation on the structural, optical, and ferroelectric properties of (Bi:BT) ceramics. X-ray and Raman studies confirmed that both samples exhibited a single tetragonal phase before and after gamma irradiation. The Raman study showed a shift in Raman modes towards lower wavenumbers after gamma irradiation. The morphological study revealed a nonuniform grain size, with holes found after gamma irradiation. The gamma radiation-induced combined effect was proved by a detailed analysis of bond lengths, angles, and octahedral distortions. After gamma irradiation, the UV–vis study indicated a decrease in the bandgap from 3.14 to 2.80 eV and a significant increase in visible light absorption. We discuss the reasons behind the decline in bandgap based on three factors: oxygen vacancies, charge compensation, and octahedral distortion. The P–E hysteresis loop study demonstrated that the ferroelectric properties could be maintained after gamma irradiation with a slight decrease in remnant polarization. The behaviour of the P–E loop was correlated with increasing gamma dose in the investigated ceramics, demonstrating a solid gamma dependence in the loops’ profile. We guess that the results obtained on the sample after gamma irradiation may be promising for electronic device applications.

## Figures and Tables

**Table 1:** Parameter estimates from Rietveld refinement for Bi:BT ceramic before and after gamma irradiation at room temperature.

Sample/Parameters		@0 Gy	@400 Gy	@800 Gy	@1000 Gy
Crystal structure		Tetragonal	Tetragonal	Tetragonal	Tetragonal
Lattice constant a = b (Å)		3.99802	3.98215	3.9936	4.0111
c (Å)		4.02460	4.0199	4.0196	4.0318
c/a		1.00664	1.00948	1.00651	1.005161
V (Å)^3^		64.329864	64.34994	64.52533	65.20208
Space group		P4mm	P4mm	P4mm	P4mm
Space group number		99	99	99	99
Theoretical density g/cm^3^		6.19	6.073	6.103	6.106
Measured density g/cm^3^		5.881	5.741	5.718	5.588
Relative density (%)		95	94.5	93.7	91.5
Porosity (%)		5	5.5	5.3	8.5
Distortion crystal lattice (a)		-	−0.00397	0.00288	0.00438
Distortion crystal lattice (c)		-	−0.00117	−7.46 × 10^−5^	0.00304
Crystallite size (nm)		105.59	59.62	29.95	31.110
Lattice strain		0.0010	0.0018	0.0036	0.0035
Ba/Bi	x	0.00000	0.00000	0.00000	0.00000
	y	0.00000	0.00000	0.00000	0.00000
	z	0.00000	0.00000	0.00000	0.00000
	Occ	1.000	1.000	1.000	1.000
	Site	1a	1a	1a	1a
	Sym	4 mm	4 mm	4 mm	4 mm
Ti/Co	x	0.50000	0.50000	0.50000	0.50000
	y	0.50000	0.50000	0.50000	0.50000
	z	0.52430	0.53082	0.52376	0.52835
	Occ	1.134	1.295	0.97340	0.96697
	Site	1b	1b	1b	1b
	Sym	4 mm	4 mm	4 mm	4 mm
O1	x	0.50000	0.50000	0.50000	0.50000
	y	0.50000	0.50000	0.50000	0.50000
	z	0.03260	0.05652	0.03152	0.05517
	Occ	0.872	1.371	1.23106	1.34734
	Site	1b	1b	1b	1b
	Sym	4 mm	4 mm	4 mm	4 mm
O2	x	0.50000	0.50000	0.50000	0.50000
	y	0.00000	0.00000	0.00000	0.00000
	z	0.48920	0.51915	0.48851	0.61300
	Occ	1.191	1.467	1.32096	1.41480
	Site	2c	2c	2c	2c
	Sym	2 mm	2 mm	2 mm	2 mm
R_p_%		10.7	4.61	4.87	5.16
R_wp_%		7.41	5.77	6.14	7.21
Rex%		6.26	5.19	5.33	6.17
χ^2^		1.4	1.23	1.32	1.36

**Table 2:** Fundamental wavenumbers (cm^−1^) of Bi:BT ceramics before and after gamma irradiation, with their symmetry modes estimated from Gaussian fitting of the Raman spectra.

Modes	@0 Gy	@400 Gy	@800 Gy	@1000 Gy
E(TO_2_)	197	195	194.5	194.12
A1(TO_2_)	245	244	243.8	243.6
E(TO_3_)	309	308	307	306.6
A1(TO_3_)	512	509	508.5	506
E(LO_4_)	561	555	554	553
A1(LO_3_)	724	721	720	719

**Table 3:** Variation in bond length, bond angle, octahedral distortion, and bandgap energy with different gamma-ray doses.

Sample/Parameters	@0 Gy	@400 Gy	@800 Gy	@1000 Gy
(Ba/Bi-O1)×4 Å	2.83007 (5)	2.80775 (0)	2.8197 (3)	2.8379 (0)
(Ba/Bi-O2)×4 Å	2.86744 (4)	2.85473 (0)	2.8663 (3)	2.5370 (17)
(Ba/Bi-O2) ×4 Å	2.83007 (5)	2.74735 (0)	2.8004 (3)	2.5370 (17)
(Ti-O1) Å	1.9789 (0)	1.97964 (0)	1.9535 (3)	1.9074 (17)
(Ti-O1) Å	2.0457 (0)	2.05334 (0)	2.0461 (3)	2.1244 (17)
(Ti-O2) ×4 Å	1.9789 (5)	1.9795 (0)	1.9692 (3)	2.02944 (4)
(O2-Ti-O2) deg	177.3226 (0)	173.9156 (3)	170.8418 (13)	160.637 (4)
(O1-Ti-O1) deg	180	180	180	180
Octahedral distortion × 10^−4^	1.23712	1.505405	2.066853	9.66842
Bandgap energy (eV)	3.14	3.019	2.89	2.80

**Table 4:** Output results obtained from Cole–Cole plots fitting for the Bi:BT sample measured before and after gamma irradiation at different gamma-ray doses.

	@0 Gy	@400 Gy	@800 Gy	@1000 Gy
*R*s (Ω)	81.73	81.54	53.38	23.44
*Q*1 (Fs^n^)	5.127 × 10^−5^	6.877 × 10^-5^	2.479 × 10^-5^	5.968 × 10^-5^
*R_g_* (k Ω)	620.7	578.4	367.3	389.8
*Q*2 (Fs^n^)	3.654 × 10^-7^	8.694 × 10^-8^	5.649 × 10^-7^	5.117 × 10^-6^
*R_gb_* (k Ω)	1759.6	1508.7	1522.9	1505.9

## Data Availability

Data are contained within the article.
